# Management of Ischemic Stroke in a Tertiary Care Hospital in Khyber Pakhtunkhwa: Existing Status and Prospective Opportunities

**DOI:** 10.7759/cureus.63094

**Published:** 2024-06-25

**Authors:** Zafar Mehmood, Sami Ullah Khan, Bilal Khan, Syed Mansoor Shah, Abdullah Khan, Hubab Ali, Syed Jawad Ahmed, Muneeb Ur Rehman, Hina Nawaz, Riaz Uliqbal

**Affiliations:** 1 Neurology, Lady Reading Hospital-Medical Teaching Institute (LRH-MTI), Peshawar, PAK; 2 Internal Medicine, Ayub Teaching Hospital, Abbotabad, Abbotabad, PAK; 3 Neurosurgery, Lady Reading Hospital-Medical Teaching Institute (LRH-MTI), Peshawar, PAK; 4 Neurosurgery, Queen Elizabeth Hospital Birmingham, University Hospitals Birmingham NHS Foundation Trust, Birmingham, GBR; 5 Clinical Neurosciences, Royal Infirmary, Edinburgh, GBR; 6 Radiology, Lady Reading Hospital-Medical Teaching Institute (LRH-MTI), Peshawar, PAK

**Keywords:** iv thrombolysis, door-to-needle time, door-to-ct timings, pre-hospital delay, ischemic stroke

## Abstract

Background and objective

Khyber Pakhtunkhwa is the third largest province of Pakistan by population and has a high incidence of ischemic stroke. We evaluated all patients who presented to the largest tertiary care facility in the province to learn about the current trends in the management of ischemic stroke and explore future opportunities in this regard.

Materials and methods

This prospective observational research was carried out at the Lady Reading Hospital-Medical Teaching Institute (LRH-MTI), Peshawar, in the province of Khyber Pakhtunkhwa (KP). The hospital's ethics committee granted the required permissions for the research. Any patient with an ischemic stroke diagnosis, regardless of age, met the inclusion criteria if their diagnosis was confirmed by clinical assessment, imaging (such as CT or MRI), or both. The clinical parameters that were evaluated included the duration since the patient's reported onset of symptoms, the patient's first mode of transfer to the hospital (such as ambulance, private vehicle, or other means), and the date and time of admission to the hospital. A structured database containing the data was utilized, and IBM SPSS Statistics for Windows, Version 25 (released 2017; IBM Corp., Armonk, New York, United States) was used for statistical analysis.

Results

One hundred fifty-six stroke patients were diagnosed throughout the study period, with 76 of them having an ischemic stroke, accounting for 49% of all stroke cases. Approximately 43% (n = 33) of the patients were from Peshawar, with the remaining patients coming from adjacent districts. There was only a small percentage (19%, n = 15) of patients who were eligible for any thrombolytic therapy, and the majority (93%) were brought by private vehicles. There was a significant association between age and arrival in the emergency room (p = 0.003).

Conclusion

The study reveals subpar ischemic stroke management in Khyber Pakhtunkhwa, requiring coordinated efforts, modernization of treatment methods, and increased public awareness to improve patient outcomes.

## Introduction

Stroke is the second-most common cause of morbidity and death worldwide. It can be caused by a blood vessel rupturing, resulting in the formation of a hematoma and pressure effects on the brain, as in the case of a hemorrhagic stroke, or it can be caused by occlusion of blood vessels, leading to reduced blood flow to the brain and functional disability or death [1]. Geographical location and population can affect the prevalence of these types of strokes; in developing countries, ischemic stroke cases can be as low as 66%, while in developed countries, they can reach up to 90% of all stroke cases [2]. This discrepancy may result from various factors, such as variations in the healthcare infrastructure and accessibility to medical services [2]. Stroke has a 5.5 million annual death rate, but its effects go beyond death; due to high morbidity, up to 50% of survivors experience long-term disability. Stroke has major public health implications as well as economic and social repercussions. Current data shows that there are nearly 25.7 million stroke survivors, 6.5 million stroke deaths, and 113 million disability-adjusted life years (DALYs) lost due to stroke [3,4]. Though historically common in developed countries, the incidence of stroke has decreased there as a result of efficient preventive measures. On the other hand, the burden on the developing world is currently increasing, made worse by changes in health and demographics [3,5]. The main goals of stroke management are risk factor reduction and the adoption of a healthy lifestyle as primary preventative measures. Additionally, it aims to prevent secondary brain injury by implementing measures to reperfuse the ischemic brain [5]. In Pakistan, stroke incidence is poorly documented due to the absence of comprehensive epidemiological studies. However, community-based research from Karachi shows a high lifetime risk for stroke and transient ischemic attacks (TIAs) [6,7]. A local community-based study of around 23000 individuals showed that there was about 1.2% prevalence in Khyber Pakhtunkhwa [8]. Further to add, stroke management often depends on aspirin in emergencies, which is suboptimal, and challenges include delayed access to adequate stroke care, limited options for interventional therapy, and insufficient training for physicians in acute stroke management [7].

In the current study, conducted in an esteemed tertiary care facility in Khyber Pakhtunkhwa, Pakistan, we evaluated the treatment of ischemic stroke in light of the 2019 Acute Ischemic Stroke (AIS) guideline updates [9]. The study's objectives were to give a basis for analyzing the region's current ischemic stroke management strategies, provide insights into possible advancements and future directions, and execute a comparative analysis with global standards and practices to enable a thorough understanding of the opportunities and challenges unique to the region in managing ischemic strokes.

## Materials and methods

Study design and settings

The purpose of this prospective observational study was to collect data on ischemic stroke patients at the Lady Reading Hospital-Medical Teaching Institute (LRH-MTI), Peshawar, in the Pakistani province of Khyber Pakhtunkhwa (KP). Data was collected between October 2023 and March 2024, and the research complied with ethical standards and protected patient privacy at all times, as approved by the ethics committee of the hospital. Using Openepi®, it was determined that 64 people would make up the sample size for a population of one million with a 0.8% prevalence (i.e., 70% of all 1.2% strokes in the area are ischemic strokes) and a 97% confidence interval.

Data collection procedure

Regardless of age, any patient diagnosed with an ischemic stroke met the inclusion criteria, provided that imaging in the form of computed tomography (CT) or magnetic resonance imaging (MRI), or clinical assessment confirmed the diagnosis. Patients with hemorrhagic strokes were excluded from the study. Additionally, patients who were excluded had inadequate medical records or lacked important data points. The patient's information was entered into a form, which included the patient's age (in years), gender (marked as either male or female), city or residential area, and level of education attained. Clinical parameters that were evaluated included the duration since the patient's reported onset of symptoms, the patient's first mode of hospital transfer (e.g., ambulance, private vehicle, or other means), the date and time of hospital admission, the interval between admission and the CT scan, the door-to-needle time (the time between hospital arrival and the initiation of any therapeutic intervention, e.g., thrombolysis or anticoagulation), the door-to-full authorized floor/admission time, and any associated risk factors (e.g., diabetes, hypertension, prior history of stroke, non-compliance with medication, etc.). Patients who were suspected of having a stroke were tracked until the completion of their CT scans, and if an ischemic stroke was discovered, they were added to the study. An on-call doctor who was tasked with interviewing the patients' companions using a pre-made form gathered the data.

Analysis of the data

The data were stored in a structured database, and statistical analysis was performed using IBM SPSS Statistics for Windows, Version 25 (released 2017; IBM Corp., Armonk, New York, United States). For relevant variables, descriptive statistics like mean, standard deviation, and frequency were calculated. In order to look into relationships and differences, inferential statistics such as t-tests and chi-square tests were utilized.

## Results

One hundred fifty-six stroke patients were diagnosed throughout the study period, with 76 of them having an ischemic stroke, accounting for 49% of all stroke cases. There were 30 males and 46 females, with a male-to-female ratio close to 1:1.5. The mean age was 62.24 years, with a standard deviation of 13.88, ranging from 23 to 90 years. Approximately 43% of the patients were from Peshawar, with the remaining patients coming from adjacent districts such as Mardan, Charsadda, Khyber, and Bajaur Districts. A small number of patients (n = 5, 6.5%) were from Afghanistan. The demographics and details of patient characteristics and various variables and their relative percentages and numbers are outlined in Table 1.

**Table 1:** Shows the patient characteristics and the relative percentage of various variables TIA: transient ischemic attack; NIHSS: National Institute of Health Stroke Scale; CT: computed tomography

Variable	Subgroup	Number of patients	Percentage
Gender	Male	30	39.5
	Female	46	60.5
Age	<60 years	21	27.6
	>60 years	55	72.4
District of residence	Peshawar	33	43.4
	Other	43	56.6
Education level	Nil	10	13.2
	Primary	49	64.5
	High School	15	19.7
	College	2	2.6
Mode of transfer	Private taxi/car	59	77.6
	Private ambulance	11	14.5
	Rescue 1122	6	7.9
Previous stroke or TIA	No	35	46.1
	Yes	41	53.9
NIHSS score at presentation	Mild (1-5)	14	18.4
	Moderate (6-11)	23	30.3
	Severe (12-18)	21	27.6
	Very severe (19-)	18	23.7
Time since last seen normal	0-4.5 hours	15	19.7
	4.5-24 hours	13	17.1
	>24 hours	47	61.8
	Wake-up stroke	1	1.3
Door-to-CT time	<30 minutes	32	42.1
	30 minutes-1 hour	26	34.2
	1-3 hours	11	14.5
	>3 hours	7	9.2
Door-to-first response time	<30 minutes	28	36.8
	30 minutes-1 hour	27	35.5
	1-3 hours	15	19.7
	>3 hours	6	7.9
Door-to-admission time	<30 minutes	7	9.2
	30 minutes-1 hour	20	26.3
	1-3 hours	19	25.0
	>3 hours	30	39.5

All patients underwent CT scans, but only 33% (n = 25) underwent an MRI. Strokes affected the anterior circulation in 55 patients (72.36%), both the anterior and posterior circulation in seven patients (9.21%), and the posterior circulation in 14 patients (18.42%). The duration of the stroke was calculated using the patient's last observed normal condition as a reference; only 13 (17.1%) of the total patients had a stroke occurring between 4.5 to 24 hours, while 19% (n = 15) had one stroke in less than 4.5 hours. The majority of these patients (n = 47, 61.8%) had a stroke lasting more than 24 hours, and only one wake-up stroke was observed. Neurosurgical interventions were done in only two patients (2.6%) in the form of decompressive craniectomy. Door-to-CT, door-to-first response, and door-to-admission time were assessed in the form of a duration of fewer than 30 minutes to more than three hours and were grouped into four categories. 

Risk factors were found in almost all patients depending on the different etiologies, and these included hypertension, diabetes, previous history of stroke, family history of stroke, and non-compliance with medications. To find any association between the various variables and the reason for the delay, Fisher’s exact test was used, and there was a significant association between age and the arrival to the emergency room (p = 0.003), with no clear benefit to the outcome assessment noted. However, a small percentage were eligible for any therapeutic intervention, which was limited to intravenous alteplase but was not provided during the study period due to some reasons; no mechanical thrombectomy was available in the facility. The results of the dependent variable (last seen normal until arrival at the ER) and other independent variables are shown in Table 2.

**Table 2:** Shows the association and results of various factors that influence the dependent factor, i.e., time since last seen till arrival in the emergency room (ER) NIHSS: National Institute of Health Stroke Scale

		Time from last seen normal till arrival to ER, n (%)					p
		0-4.5 Hours	4.5-24 Hours	More than 24 hours	Wake up stroke	Total (n)	
Gender	Female	9 (60)	10 (74.9)	27 (57.4)	0 ()	46	0.371
	Male	6 (40)	3 (20.1)	20 (42.6)	1 (100)	30	
Age limit in years	<60	9 (60)	5 (38.5)	7 (14.9)	0 (0)	21	0.003
	>60	6 (40)	8 (61.5)	40 (85.1)	1 (100)	55	
District	Same	8 (53.3)	5 (38.5)	19 (40.4)	1 (100)	33	0.596
	Other	7 (46.7)	8 (61.5)	28 (59.6)	0 ()0	43	
Education	No Education	0 (0)	2 (15.4)	8 (17.0)	0 (0)	10	0.500
	Primary level	13 (86.7)	7 (53.8)	28 (59.6)	1 (100)	49	
	High School	2 (13.3)	3 (23.0)	10 (21.3)	0 (0)	15	
	College	0 (0)	1 (7.6)	1 (2.1)	0 (0)	2	
Mode of transfer	Private car/taxi	12 (80)	10 (74.9)	36 (76.6)	1 (100)	59	0.842
	Private ambulance	(13.2)	3 (23.1))	6 (12.8)	0 (0)	11	
	Rescue 1122	1 (6.6)	0 (0)	5 (10.6)	0 (0)	6	
Previous stroke	No	10 (66.6)	8 (61.5)	17 (36.1)	0 (0)	35	0.069
	Yes	5 (33.4)	5 (38.5)	30 (63.9)	1 (100)	41	
NIHSS score	Mild	5 (33.3)	2 (15.4)	7 (14.9)	0 (0)	14	0.085
	Moderate	6 (40.0)	6 (46.1)	11 (23.4)	0 (0)	23	
	Severe	4 (26.7)	3 (23.0)	14 (29.8)	0 (0)	21	
	Very severe	0 (0)	2 (15.4)	15 (31.5)	1 (100)	18	

## Discussion

Stroke is a major global issue, responsible for over 30% of deaths [1]. It is particularly worrisome in developing nations, occurring at a younger age with little impact on stroke outcomes [1,9,10]. There is a concerning rise in the occurrence of hemorrhagic stroke compared to ischemic stroke in emerging countries [10]. Ischemic stroke accounted for 49% of our research population. This is concerning because the reported incidence of ischemic stroke in wealthy countries is usually 80-90% of all stroke patients, whereas it is approximately 60-70% in African countries [1,10,11]. To determine the frequency and percentage of ischemic strokes in the population, extensive studies are needed. The male-to-female ratio in our study was approximately 1:1.5, meaning that there were more females than males in the study group. Our study, with its small sample size, found no significant association between gender and ischemic strokes, which is consistent with a significant prevalence study in the area [1,4,8,10], despite the majority of studies showing a slight female predominance. Our sample's mean age was 62.24 ± 13.88 standard deviation (SD), higher than the average age of 43.39 ± 0.84 years in a large population-based study and 48.7 ± 12.8 SD in another relatively small community-based study [8,9]. These two investigations' findings, however, revealed lower average ages. The average age of a stroke is under 60 years in developing countries and over 75 years in industrialized countries [1,2]. Patients are referred to our hospital primarily from throughout the province and neighboring countries. Of the total patients, 33 (43.4%) were from within the district, and 43 (56.6%) were from outside, including five (6.6%) patients from Afghanistan. Most people (n = 59, 77.6%) took a taxi or their own car to get to the main facility. The results of Fisher's exact test (p = .217) showed that there was no significant correlation between the mode of transportation and the residential district, whether from Peshawar or another district. Prehospital care from emergency medical services is considered essential in developed nations; in fact, the emergency medical team treats 36-42% of acute stroke patients initially [12]. The government ambulance service transported 7.9% of patients, while the private ambulance service transported 14.5%. However, most of the medical staff in both services is not well-trained or knowledgeable about strokes. The only thing that sets private ambulances apart from private taxis is the oxygen supply; these vehicles are driven by individuals without training in medicine. A far better option than the other two in our setup, Rescue 1122 is a state-owned ambulance service run by first-aid medical professionals.

Most patients in our cohort had an education level below primary (n = 59, 77.8%), while just a small number had a college-level education (n = 2, 2.6%). In addition to making people more aware of diseases and their own health than people with lower levels of education, education also improves understanding of health conditions, which makes educated people feel better about the environment than uneducated people [13]. Education is a powerful tool for raising awareness of health issues like stroke and promoting behavioral changes in this area. Children, teenagers, and the general public may change their behavior as a result of this. Stroke incidence and death rates can be eventually decreased by educating people, particularly adolescents, about the symptoms and risks of stroke and how to prevent them. This can also help with early detection and timely hospital visits [13,14].

Most notably, a small percentage of patients (n = 15, 19.7%) met the criteria of having visited the hospital within 4.5 hours of the onset of their symptoms. This is concerning because there are relatively few patients who arrive at the hospital within the necessary window to receive medication intervention in a country where stroke incidence and complications are high. Data from the National Health Service (NHS) showed that the percentage of stroke victims who reached the hospital within the designated time frames was 40.2% within three hours of symptom onset, 51.6% within 4.5 hours, and 59.14% within six hours; furthermore, the data from the month of August 2023 showed that the average time for ambulance patients suffering from a stroke from a 999 emergency call to hospital arrival was one hour and 31 minutes, which was a decrease of more than 17 minutes from the previous year, showing an improved trend [15]. However, there are regional variations in the percentage of ischemic stroke patients who arrive at the hospital within 4.5 hours, as a study carried out in a North Indian tertiary care hospital revealed that 36.17% of acute ischemic stroke patients arrived within the 4.5-hour window and were deemed eligible for thrombolysis [16]. According to the study, 91% of acute stroke patients in Uganda presented to the emergency room after three hours, indicating a significant delay; only 9% of acute stroke patients reached the tertiary center within three hours of the onset of symptoms [17]. In contrast, 92.2% of patients arrived at the hospital within three hours of the onset of symptoms, according to a Taiwanese study [18]. Only 40% (6/15 patients) of those who arrived at the hospital in less than 4.5 hours had door-to-CT times of less than 30 minutes, according to a more thorough analysis of our patient population. This figure reflected the fact that only 8% of the first 19.7% of patients who arrived in the 4.5-hour window had their CT scans completed in less than 30 minutes. Additionally, there were other significant delays in the hospital; only 28 (36.8%) patients had a door-to-first response time window of less than 30 minutes, and only seven (9.2%) patients had a door-to-specialized floor time window of less than 30 minutes in our study. The Tainwanese study showed that the median time from arrival to the emergency department to neurologic consultation and having a CT scan done was 10 and 17 minutes, respectively [18]. One reason for the delay could be that the hospital does not have a dedicated stroke team that treats these patients according to a set protocol and efficiently oversees the process. The hospital used only intravenous anti-thrombolytics for some time and does not have the facility of mechanical thrombectomies. Intravenous altepase therapy could have been administered to this small group but was discontinued during the duration of the study due to many issues. Studies have shown that IV thrombolytic therapy significantly improves the prognosis for patients with ischemic stroke. It is currently administered to approximately 16-30% of eligible patients in various countries, and its use has increased by up to 7% annually [17-19].

There are a few limitations that could make interpreting our findings challenging. Because of its small sample size and concentration on a single hospital, the study's findings cannot be broadly applied; nonetheless, the hospital continues to be the largest tertiary care facility in the area and represents the health and well-being of its residents. Furthermore, we lacked a precise method to determine the onset of symptoms. This disparity could affect our understanding of prehospital delays. Other unresearched influencing factors include living arrangements, financial status, transportation access, prior medical history, justifications for treatment delays, initial hospital visits, prescribed drugs, facility conditions, and radiological tests performed. However, the results might only be relevant in our area because there are not any public hospitals in our area that currently provide IV thrombolytic therapy, and the program was started but discontinued a while back because of a number of problems. A thorough, multicenter study is necessary to understand the nature of the disease and develop a recommendation. But still, this preliminary data highlights several areas that require attention and development to improve stroke management in Khyber Pakhtunkhwa. Specifically, there are notable delays at the prehospital and hospital levels. To combat this, a policy statement should be outlined, and public awareness of stroke should be raised. For this reason, the government's primary means of disseminating information about stroke and self-care should be print, electronic, and social media channels. By putting together a specialized team, the hospital will be able to provide better inpatient care and avoid delays. To enhance stroke care and guarantee that more patients reach the hospital by the deadline, it is imperative to establish specialized stroke units, guarantee the availability of IV alteplase, and provide extra procedures, like mechanical thrombectomy, to patients who arrive later. This will increase the proportion of benefit recipients among patients, reduce the duration between patient arrivals, and extend the treatment window.

## Conclusions

The primary tertiary care facility's study results indicate that Khyber Pakhtunkhwa's management of ischemic stroke has been subpar on many levels. Few ischemic stroke patients were able to reach the hospital in time to be eligible for treatment; additional delays occurred at the hospital level as a result of the absence of a specialized stroke team, IV alteplase, and a facility for mechanical thrombectomy. This calls for coordinated efforts, as well as the creation and implementation of a policy at the hierarchy level to modernize stroke treatment methods. This will also increase public awareness and introduce stroke interventions.
